# Fatal Outcome of Chikungunya Virus Infection in Brazil

**DOI:** 10.1093/cid/ciaa1038

**Published:** 2020-08-07

**Authors:** Shirlene Telmos Silva de Lima, William Marciel de Souza, John Washington Cavalcante, Darlan da Silva Candido, Marcilio Jorge Fumagalli, Jean-Paul Carrera, Leda Maria Simões Mello, Fernanda Montenegro De Carvalho Araújo, Izabel Letícia Cavalcante Ramalho, Francisca Kalline de Almeida Barreto, Deborah Nunes de Melo Braga, Adriana Rocha Simião, Mayara Jane Miranda da Silva, Rhaquel de Morais Alves Barbosa Oliveira, Clayton Pereira Silva Lima, Camila de Sousa Lins, Rafael Ribeiro Barata, Marcelo Nunes Pereira Melo, Michel Platini Caldas de Souza, Luciano Monteiro Franco, Fábio Rocha Fernandes Távora, Daniele Rocha Queiroz Lemos, Carlos Henrique Morais de Alencar, Ronaldo de Jesus, Vagner de Souza Fonseca, Leonardo Hermes Dutra, André Luiz de Abreu, Emerson Luiz Lima Araújo, André Ricardo Ribas Freitas, João Lídio da Silva Gonçalves Vianez Júnior, Oliver G Pybus, Luiz Tadeu Moraes Figueiredo, Nuno Rodrigues Faria, Márcio Roberto Teixeira Nunes, Luciano Pamplona de Góes Cavalcanti, Fabio Miyajima

**Affiliations:** 1 Federal University of Ceará, Fortaleza, Brazil; 2 Central Public Health Laboratory of Ceará State, Fortaleza, Brazil; 3 Virology Research Center, University of São Paulo, Ribeirão Preto, Brazil; 4 Department of Zoology, University of Oxford, Oxford, United Kingdom; 5 Department of Research in Virology and Biotechnology, Gorgas Memorial Institute of Health Studies, Panama City, Panama; 6 Faculdade de Medicina do Centro Universitário Christus, Fortaleza, Ceará, Brazil; 7 Death Verification Service Dr Rocha Furtado, State Health Secretariat of Ceará, Fortaleza, Ceará, Brazil; 8 Evandro Chagas Institute, Ministry of Health, Ananindeua, Brazil; 9 Universidade Federal de Minas Gerais, Brazil; 10 Ministry of Health, Brasilia, Brazil; 11 Faculdade de Medicina São Leopoldo Mandic, Campinas, São Paulo, Brazil; 12 Department of Infectious Disease Epidemiology, Imperial College London, London, United Kingdom; 13 Oswaldo Cruz Foundation (Fiocruz), Branch Ceará, Eusebio, Brazil

**Keywords:** chikungunya virus, *Alphavirus*; arthritogenic, arbovirus, fatal cases

## Abstract

**Background:**

Chikungunya virus (CHIKV) emerged in the Americas in 2013 and has caused approximately 2.1 million cases and >600 deaths. A retrospective investigation was undertaken to describe clinical, epidemiological, and viral genomic features associated with deaths caused by CHIKV in Ceará state, northeast Brazil.

**Methods:**

Sera, cerebrospinal fluid (CSF), and tissue samples from 100 fatal cases with suspected arbovirus infection were tested for CHIKV, dengue virus (DENV), and Zika virus (ZIKV). Clinical, epidemiological, and death reports were obtained for patients with confirmed CHIKV infection. Logistic regression analysis was undertaken to identify independent factors associated with risk of death during CHIKV infection. Phylogenetic analysis was conducted using whole genomes from a subset of cases.

**Results:**

Sixty-eight fatal cases had CHIKV infection confirmed by reverse-transcription quantitative polymerase chain reaction (52.9%), viral antigen (41.1%), and/or specific immunoglobulin M (63.2%). Co-detection of CHIKV with DENV was found in 22% of fatal cases, ZIKV in 2.9%, and DENV and ZIKV in 1.5%. A total of 39 CHIKV deaths presented with neurological signs and symptoms, and CHIKV-RNA was found in the CSF of 92.3% of these patients. Fatal outcomes were associated with irreversible multiple organ dysfunction syndrome. Patients with diabetes appear to die at a higher frequency during the subacute phase. Genetic analysis showed circulation of 2 CHIKV East-Central-South African (ECSA) lineages in Ceará and revealed no unique virus genomic mutation associated with fatal outcome.

**Conclusions:**

The investigation of the largest cross-sectional cohort of CHIKV deaths to date reveals that CHIKV-ECSA strains can cause death in individuals from both risk and nonrisk groups, including young adults.

## Teaser

The introduction of the East-Central-South African genotype of Chikungunya virus (CHIKV) in Brazil caused neurological infections that led to death. The risk of death during acute and subacute CHIKV infection was higher in patients with diabetes.

Chikungunya virus (CHIKV) is an enveloped, single-stranded positive-sense RNA virus that belongs to the *Alphavirus* genus, Togaviridae family [1]. It is mainly transmitted to humans by the *Aedes aegypti* and *Aedes albopictus* mosquitoes. Most cases are characterized by an acute infection with fever, myalgia, exanthema, and arthralgia lasting up to 3 weeks postinfection [2, 3]. For some CHIKV cases, arthralgia can persist for >3 months, indicating a transition to a chronic stage [2, 3].

In July 2014, the first autochthonous chikungunya cases were reported in Brazil. Genetic analysis revealed the co-circulation of 2 distinct genotypes introduced almost simultaneously in the country. CHIKV Asian genotype was detected in Amapá state, North Brazil and the CHIKV East-Central-South African (ECSA) genotype was first reported in Bahia state, Northeast [4]. Until December 2019, >800 000 CHIKV cases were notified in all regions of Brazil [5].

Between 2013 and 2019, the Pan American Health Organization reported 631 deaths associated with CHIKV infection in South America, likely an underestimation of deaths caused by CHIKV [6]. The highest number of CHIKV-related deaths in the Americas, 214 deaths, was reported in the Ceará state, Northeast Brazil, where an Arbovirus Death Investigation Committee was created due to the high mortality rate and increase of suspected arbovirus-associated deaths [6–8]. This high number of reported fatalities is comparable to what was observed in La Reunion Island [9, 10], where the outbreak was caused by the CHIKV Indian Ocean lineage [11]. In addition, neurologic infections and fatal cases were previously reported during the CHIKV epidemic in 1963–1965 in India [12–14].

Despite their importance for public health management, epidemiological and clinical investigations of fatal cases caused by CHIKV typically focus on case reports [15–21]. Moreover, no information exists on the genetic diversity of CHIKV circulating in Ceará state. This study summarizes the demographic, clinical, laboratorial, and postmortem findings of the largest cross-sectional cohort of CHIKV deaths to date.

## MATERIALS AND METHODS

### Study Population and Ethics Statement

We selected all fatal cases with clinical suspected arboviral infection and sera, cerebrospinal fluid (CSF), and tissue samples available recorded by the Death Verification Service of State Health Secretariat of Ceará, between December 2016 and October 2017. In each case, autopsies were undertaken within 12 hours postmortem. Sera, CSF, and tissue samples were collected and stored at −80°C for subsequent investigation. Tissues were also conserved in formalin-fixed blocks, and were sent to Evandro Chagas Institute and the Brazilian Ministry of Health for arboviral histopathological and immunohistochemistry (IHC) analyses. The study was conducted after approval by the ethics committee of the Federal University of Ceará (CAAE number: 85921418.3.0000.5054), Brazil.

### Case Definitions

A suspected fatal case of CHIKV infection was defined as a patient that presented with fever, rash, arthralgia, headache, and/or myalgia during the hospitalization or perimortem period. A confirmed CHIKV death was defined as a suspected fatal case associated with CHIKV infection, plus 1 positive laboratory result for CHIKV either by reverse-transcription quantitative polymerase chain reaction (RT-qPCR), immunoglobulin M (IgM) detection, and/or IHC [5]. In addition, we defined an acute fatal outcome as a case that lasted up to 20 days, representing the acute disease; and a subacute fatal outcome, as a case that lasted longer than 20 days up to 90 days. Patients with neurologic manifestations (eg, confusion and syncope) and RT-qPCR or IgM detection against CHIKV in CSF were considered to have a central nervous system infection caused by CHIKV [5].

### Laboratory Testing

RNA from CSF and brain tissue was extracted using the QIAamp Viral RNA Mini Kit (Qiagen) and the ReliaPrep RNA Miniprep Systems (Promega), respectively. Extracted RNA was tested by specific RT-qPCR for CHIKV [22], DENV [23], and ZIKV [24]. We also tested the postmortem sera and CSF samples using IgM-capture enzyme-linked immunosorbent assays (ELISAs) for antibody response against CHIKV (Euroimmun), DENV (Serion), and ZIKV (NovaTec Immundiagnostica GmbH). In addition, an ELISA for DENV-NS1 antigens (Bio-Rad Laboratories) was used. We also performed IHC analysis of liver and brain tissue samples of all patients [25].

Based on the laboratory diagnosis from the investigation of fatal cases of CHIKV, complete clinical records were obtained from the laboratory management system (Gerenciador de Ambiente Laboratorial [GAL]). Autopsy records for confirmed CHIKV fatal cases were obtained from the Death Verification Service (www.saude.ce.gov.br). Demographic, epidemiological, and clinical data (including comorbidities/immunosuppression history) were extracted from GAL, autopsy records, and home visit reports with the family of patients who died.

### Epidemiological Data

Epidemiological analysis of autochthonous CHIKV cases in Ceará state was undertaken using weekly reports of suspected cases from January to December 2017. Epidemiological reports of cases were obtained from the Brazilian Ministry of Health. Incidences were calculated based on the estimated population of Ceará state in 2017, as reported by the Brazilian Institute of Geography and Statistics (www.ibge.gov.br).

### Statistical Analysis

Descriptive analyses of results are reported as frequencies, arithmetic means, and ranges (when appropriate). The Mann-Whitney *U* test performed in RStudio 1.2.1335 (www.rstudio.com), was used to compare groups with comorbidities. For the regression analysis, the outcome variable was classified as patients with fatal outcome during the acute or subacute phase of the disease. The most frequent comorbidities in our cohort were used for statistical analysis. To determine comorbidities that may affect the frequency of death during the acute or subacute phase of the disease, we conducted a univariate logistic regression analysis, followed by a multivariable logistic regression controlling for sex and age. *P* values with α <.05 were considered to be significant. Statistical analyses were performed with the Stata version 14 software (StataCorp).

### Viral Genomic Sequencing and Phylogenetic Analysis

Genome sequencing was performed using a targeted multiplex PCR scheme that can amplify both CHIKV genotypes circulating in Brazil [26]. This was followed by Nanopore genome sequencing using the MinION sequencing platform (Oxford Nanopore Technologies) [26]. Preliminary runs showed that all sequences belonged to the same genotype. To improve consensus sequencing coverage, we selected 36 publicly available full genome strains and redesigned CHIKV specific sequencing primers for the genotype identified on preliminary runs by correcting mismatches. Consensus sequences were generated with a validated bioinformatics pipeline, considering a minimum coverage depth of 20× [26].

Newly generated CHIKV consensus sequences were aligned to 87 publicly available whole-genome sequences from Brazil using MAFFT version 7.450 [27]. A maximum likelihood (ML) phylogenetic tree was estimated with RAxML version 8 [28] using the GTR + I + γ substitution model [28]. Dated phylogenetic trees were estimated using BEAST version 1.10 under a GTR + I + γ model [29]. We used a strict molecular clock model and a Skygrid tree prior [30] that were previously determined as best-fit models [29]. Evolutionary analyses were run independently in duplicate for 50 million steps, and sampling parameters and trees every 10 000 steps. Maximum clade credibility summary trees were generated using TreeAnnotator version 1.10 [29].

### Data Availability

Protocols and the new sequencing primers are publicly available at caddecentre.org/protocols. Epidemiological data, phylogenetic trees, XMLs, and Ceará CHIKV genome sequences are available on the DRYAD repository (available on https://datadryad.org/stash/share/y0poMC_pufbi2DEAelhcOdJDOjFYlud_D5s56V-fMC8). New sequences have been deposited in GenBank with accession numbers MT877206-MT877211.

## RESULTS

**Figure 1.**
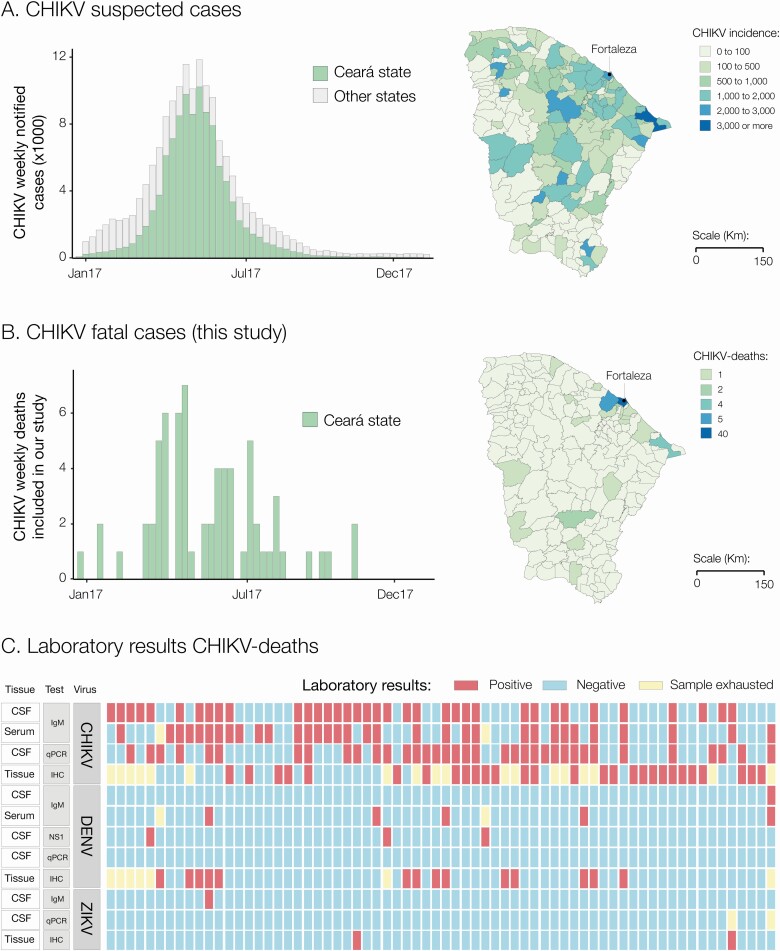
*A*, Weekly epidemiologic curve of chikungunya cases notified in Ceará state and other Brazilian states, and incidence of chikungunya cases notified by municipalities in Ceará state in 2017. *B*, Weekly epidemiologic curve and geographical distribution of chikungunya deaths described in this study. *C*, Diagnosis of 68 Chikungunya deaths described in this study. Abbreviations: CHIKV, chikungunya virus; CSF, cerebrospinal fluid; DENV, dengue virus; IgM, immunoglobulin M; IHC, immunohistochemistry; qPCR, quantitative polymerase chain reaction; ZIKV, Zika virus.

In 2017, Ceará state in Brazil experienced a major CHIKV outbreak accounting for 65.7% (n = 105 229/160 166) of suspected cases in all 27 Brazilian federal states in that year. CHIKV incidence in Ceará state was 1166 suspected cases per 100 000 inhabitants, the highest in the country [31]. Within Ceará, the municipality of Fortaleza (state capital city) accounted for the majority of CHIKV suspected cases (n = 61 825/105 229 [58.8%]) (Figure 1A). Moreover, Ceará notified a total of 194 CHIKV-related deaths in 2017 [32]. This corresponds to a case-fatality rate of 1.8 deaths per 1000 cases in 2017. Importantly, Fortaleza city reported the highest number of CHIKV-related deaths (n = 144/194 [74.2%]) in 2017 in Ceará [8]. As expected, CHIKV deaths followed a similar temporal distribution to that of the suspected cases in Ceará, with most cases reported in Fortaleza (Figure 1A and 1B).

We used RT-qPCR, serology, and IHC to ascertain the cause of death of 100 suspected arbovirus fatal cases. A total of 68% (68/100) of the cases were positive for CHIKV by at least 1 diagnostic method (Figure 1C). Of these, 70.6% (48/68) were positive by 2 or more methods. We found that 73.5% (50/68) of deaths were positive for CHIKV only, while 22% (15/68) had viral co-detection with DENV, 2.9% (2/68) co-detection with ZIKV, and 1.5% (1/68) with both DENV and ZIKV. Moreover, CHIKV-RNA was detected in the CSF of 52.9% (36/68) and in the brain of 11.1% (4/36) of the cases. Notably, no DENV or ZIKV RNA was detected in the CSF of CHIKV deaths (Figure 1C).

**Figure 2.**
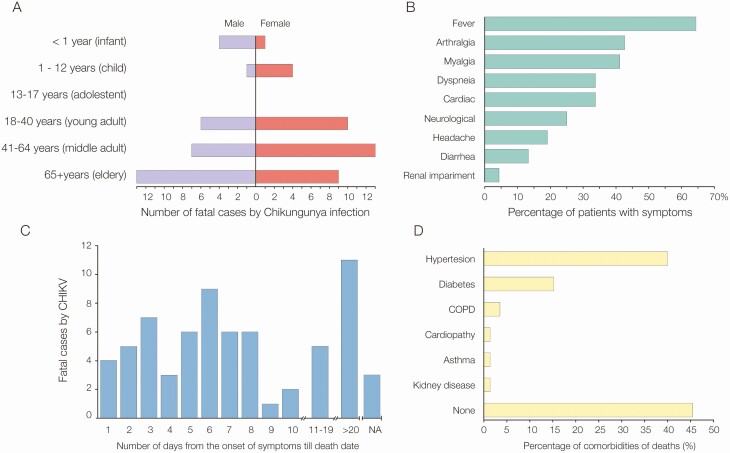
Demographics, symptoms, and comorbidities of 68 chikungunya deaths from Ceará state, Brazil. *A*, Age range and sex. *B*, Clinical characteristics. *C*, Days from the onset of symptoms of individuals till death. *D*, Comorbidities associated with chikungunya deaths. Abbreviations: CHIKV, chikungunya virus; COPD, chronic obstructive pulmonary disease; NA, not available.

Figure 2 presents an epidemiological characterization of the 68 CHIKV-confirmed deaths analyzed in this study. CHIKV deaths occurred predominantly in adults aged ≥40 years (n = 42/68 [61.8%]), with 29.4% (20/68) in middle-aged adults and 32.4% (22/68) in the elderly. The average age was 47.6 years (median, 51 years), ranging from 3 days to 85 years. Most CHIKV deaths occurred in females (n = 37/68 [54.4%]). We also report 5 CHIKV deaths in infants and 5 in children (Figure 2A).

The general clinical manifestations presented by CHIKV deaths were fever (n = 44/68 [64.7%]), arthralgia (n = 29/67 [43.3%]), cardiac symptoms such as cardiac arrest (n = 23/67 [34.3%]), dyspnea (n = 23/68 [33.8%]), diarrhea (n = 23/68 [33.8%]), neurological symptoms such as confusion and syncope (n = 17/67 [25.4%]), headache (n = 13/68 [19.1%]), and renal failure (n = 4/68 [5.9%]) (Figure 2B). The average time between the onset of symptoms and death was 12 days (range, 1–90 days). Out of the 68 CHIKV deaths, 79.4% (54/68) were patients with acute infection with fatality occurring up to 18 days from the onset of symptoms. On the other hand, 16.2% (11/68) of fatal cases were patients with subacute infection. It was not possible to obtain information on the date of symptom onset for 3 patients (4.4%). Of the 36 fatal cases with CHIKV RNA positive in CSF, 3 CHIKV deaths were patients presenting with up to 20 days of infection.

Subsequently, we analyzed comorbidity and immunosuppression records available for 65% (44/68) of CHIKV deaths. No comorbidities were reported in 45.5% (20/44) of the medical records, while 27.3% (12/44) had 1 comorbidity, 25% (11/44) had 2 comorbidities, and only 2.3% (1/44) had 3 comorbidities. The most frequent comorbidities were hypertension in 40.9% (18/44) and diabetes in 15.9% (7/44) of CHIKV deaths (Figure 2D). All patients with diabetes also had hypertension, but only 38.9% (7/18) of patients with hypertension had diabetes.

The average age of CHIKV deaths in people with comorbidities (60 years [range, 31–85 years]) differs from the average age of CHIKV deaths in those without comorbidities (37.4 years [range, 3 days–79 years]) (*P* = .003, Mann-Whitney *U* test). Multivariable logistic regression analysis, controlled for age and sex, suggests that the risk of dying during the subacute phase of CHIKV infection increases 7 times in cases with diabetes when compared to cases without diabetes (odds ratio, 7.7; *P* = .033; Table 1 and Supplementary Table 1). No statistically significant difference was observed between the days to death of patients with and without comorbidities (*P* = .2855, Mann-Whitney *U* test). No immunosuppression by cancer, human immunodeficiency virus, or corticosteroid treatment was reported in CHIKV deaths.

**Table 1.** Univariate and Multivariable Logistic Regression Analysis of the Presence of Acute or Subacute Fatalities by Chikungunya Infection

Symptoms	Risk Fatality During Subacute Disease					
	Unadjusted OR	(95% CI)	*P* Value	*φ* Adjusted OR	(95% CI)	*P* Value
Hypertension						
No	Ref	Ref	Ref	Ref	Ref	Ref
Yes	2.77	(.58–13.2)	.200	4.1	(.62–26.9)	.141
Diabetes						
No	Ref	Ref	Ref	Ref	Ref	Ref
Yes	6.93	(1.2–40.9)	**.033**	**7.7**	(1.2–50.0)	**.033**
Diabetes and hypertension						
No	Ref	Ref	Ref	Ref	Ref	Ref
Yes	1.50	(.20–10.8)	.687	2.48	(.20–29.4)	.472

*φ* adjusts by sex (OR, 0.96; *P* = .966) and age (OR, 1.00; *P* = 9.66).

Abbreviations: CI, confidence interval; OR, odds ratio. Results with p < 0.05 are shown in the bold.

**Figure 3.**
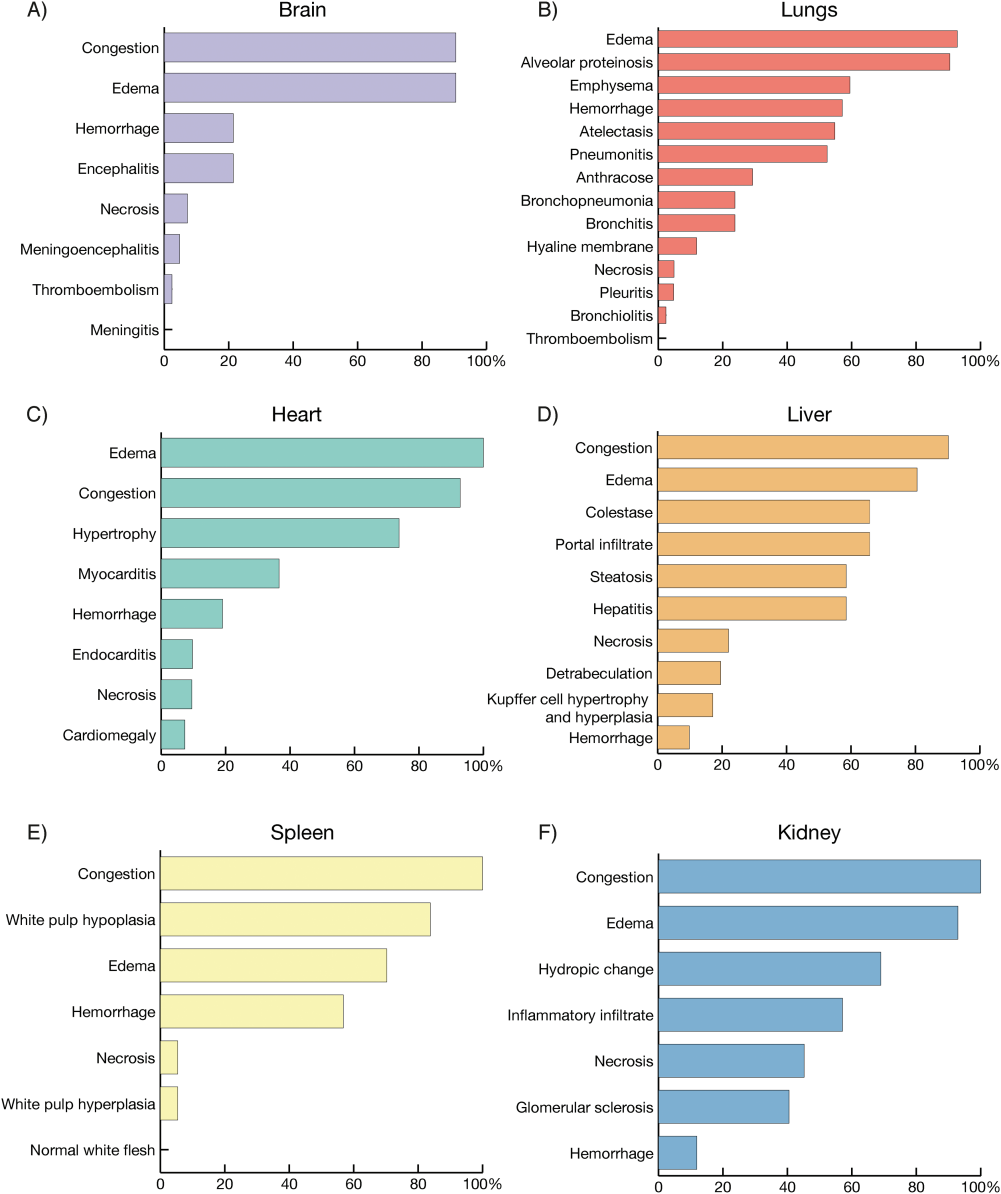
*A–F*, Autopsy findings of 42 chikungunya deaths from Ceará state, Brazil.

For 42 (61.8%) autopsied CHIKV deaths, heart and/or respiratory failure were the most frequent causes of death (76.2% [32/42]). Autopsies revealed vascular congestion and edema in main organs of CHIKV deaths (Figure 3). Also, CHIKV deaths were frequently associated with hepatitis (58.5% [24/41]), pneumonitis (52.4% [22/42]), myocarditis (36.6% [15/41]), and encephalitis (21.4% [9/42]) (Figure 3 and Supplementary Table 2). Other frequent findings in the lungs were hemorrhage in 57.1% (24/42), 54.8% (23/42) atelectasis, 33.3% (14/42) megakaryocytes, and 26.2% (11/42) hemosiderophages (Supplementary Figure 1).

**Figure 4.**
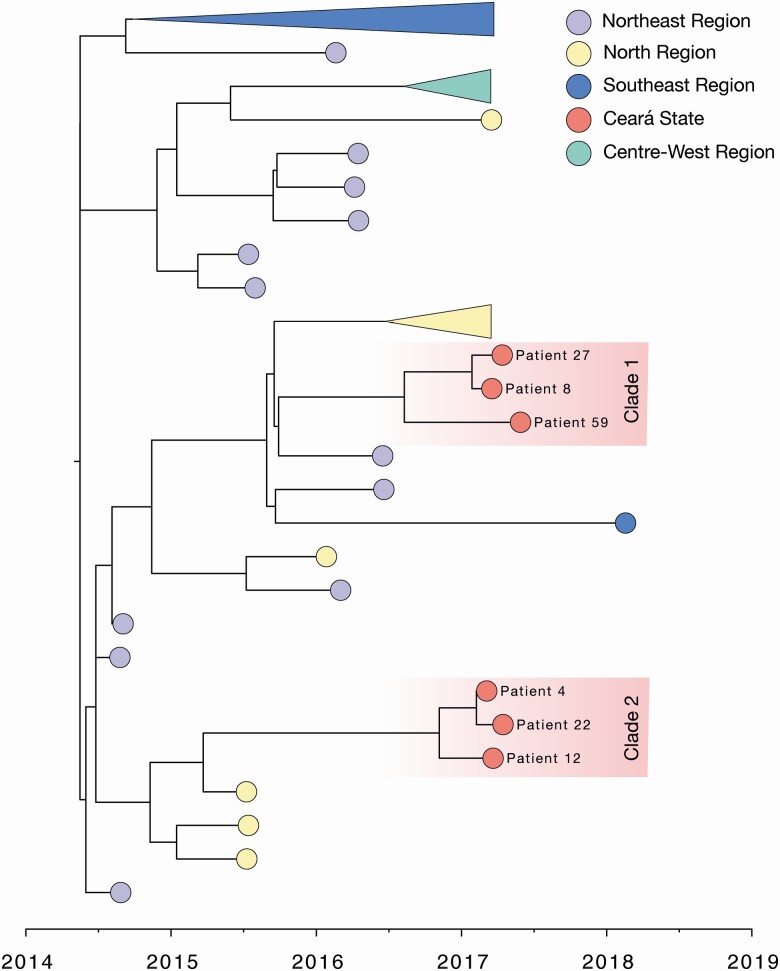
Maximum clade credibility tree of the East-Central-South African genotype of Chikungunya virus in Brazil (n = 71), including 6 new sequences from Ceará State. Tips are colored according to the source region of each sample. Clusters from the Southeast, Center-West, and North regions have been collapsed for better visualization. The 2 clusters of sequences from this study were identified as clade 1 and clade 2 based on the earliest estimated time to most recent common ancestor. A molecular clock approach was used for generating the time-rooted tree (see Methods).

To elucidate the genetic diversity of CHIKV strains in Ceará, we sequenced the viral genomes from 7 samples recovered from 6 CHIKV deaths (patients 8, 12, 22, and 27 from CSF samples; patient 59 from blood; and patient 4 from CSF and blood). ML and Bayesian phylogenies suggested that sequenced CHIKV strains formed 2 monophyletic clades (1 and 2) with maximum statistical support within the ECSA lineage circulating in Brazil (bootstrap score = 100, posterior support = 1.00). Our analyses suggest that CHIKV was introduced into Ceará state between early 2015 and mid 2016, with the most common ancestor for each cluster at around early to mid-2016 (cluster 1: 2016.60 [95% highest posterior density {HPD}, 2016.39–2016.90]; cluster 2 = 2016.84 [95% HPD, 2015.5–2016.83]) (Figure 4). Based on the analysis of the genomes obtained in this study compared to the genomes available, we did not find mutations associated with enhanced infection and transmission in mosquitoes, or increased virulence, and no unique mutations associated with the Ceará sequences.

## DISCUSSION

Herein, we perform the most comprehensive characterization with description of clinical, demographic, and laboratory findings of the largest cross-sectional study population of confirmed CHIKV deaths to date. We confirm CHIKV infection in 68 cases and exclude co-detection with ZIKV and/or DENV in 73.5% of them. Although comorbidities and older age play an important role in CHIKV deaths, we show that almost half of fatal cases did not have any comorbidities and that 38.2% (26/68) of them were aged ≤40 years. In addition, our autopsy results point to cardiac and respiratory failure, possibly due to generalized congestion and edema as the main death cause in the course of CHIKV infection.

In contrast to other arboviral diseases (eg, DENV and ZIKV), CHIKV infection is symptomatic in most individuals, manifesting as a typical rapid-onset febrile disease, characterized by intense arthralgia, myalgia, headache, and rash [2]. The clinical manifestations described herein for the 68 CHIKV deaths were consistent with previous case reports of typical CHIKV infections [2]. However, the detection of CHIKV RNA in the CSF of 36 patients and 4 brain samples and the high frequency of neurological symptoms are strongly indicative of a neurotropic role of CHIKV associated with a severe central nervous system infection in more than half of CHIKV-deaths.

CHIKV deaths have only been reported as an outcome of acute infection [15–20]. However, 16.2% of the fatal cases in our study were subacute CHIKV infection, with symptom onset within 25–90 days prior to death, showing that subacute CHIKV infections may also have a fatal outcome. These findings are consistent with studies that demonstrate that the peak of excess deaths occurs with a lag of 1 month in relation to reported cases of chikungunya [33]. Subacute CHIKV deaths especially may be underreported as their long-term duration might decrease the idea of an association between a CHIKV diagnosis and a deadly outcome, while increasing the apparent importance of comorbidities or even hospital-acquired infections as the main cause of death [6].

Severe chikungunya infection has been correlated with age dependency and follows a U-shaped parabolic curve [2]. Here, we confirmed that middle-aged adults and the elderly constituted the majority of CHIKV deaths, followed by infants and children, while no fatal cases in adolescents were identified [2]. However, 23.5% of CHIKV deaths occurred in young adults, indicating that deaths by CHIKV infection do occur at young ages more than previously anticipated. Future clinical and epidemiological studies of CHIKV could help to shed light on the risk of infection and disease severity per age class.

Previously reported risk groups for severe chikungunya infection were patients with comorbidities or those immunocompromised [2]. Our results partially support this conclusion, as 54.5% of our cases had at least 1 comorbidity. Notably, we observed that diabetes considerably increases the risk for death in the subacute phase of CHIKV infection. However, 45.5% did not have any comorbidity reported in their medical records. In addition, none of our cases had any medical history of immunosuppression. Collectively, these results suggest that chikungunya infection can lead to patient death even in the absence of an underlying medical condition.

Our autopsy and histopathological analyses suggest that multiple organ dysfunction syndrome in CHIKV infection may occur by hemodynamic disturbance (vascular congestion, edema, and hemorrhage) of main organs, predominately heart and lungs. Also, the presence of hemosiderophages and megakaryocytes within the lungs suggests a role for increasing pressure on alveolar capillaries leading to hemorrhagic complications, as previously described for DENV [34, 35]. Therefore, careful monitoring of fluid balance and administration of hypotonic solutions can be required as part of clinical management.

CHIKV has widely spread throughout Ceará state. Our genetic analysis revealed that virus strains circulating in the state belong to the CHIKV-ECSA genotype introduced in Bahia state in 2014 [4]. We found no amino acid mutations associated with enhanced infection and transmission in mosquito vectors [11].

Our results demonstrated that CHIKV-associated deaths are not a rare event during large outbreaks, and may occur even in low-risk populations (young age and no comorbidities). Thus, the total disease burden must be reevaluated considering these outcomes. Guidelines and diagnoses need to be improved to prevent fatal outcomes in CHIKV-infected patients.

## Supplementary Data

Supplementary materials are available at *Clinical Infectious Diseases* online. Consisting of data provided by the authors to benefit the reader, the posted materials are not copyedited and are the sole responsibility of the authors, so questions or comments should be addressed to the corresponding author.

ciaa1038_suppl_Supplementary-Appendix

## Notes

***Author contributions.*** S. T. S. L., L. M. S. M., F. M. C. A., I. L. C. R., M. J. M. S., C. P. S. L., R. R. B., and M. P. C. S. performed the RT-qPCR and serological tests. F. K. A. B., D. N. M. B., A. R. S., R. M. A. B. O., C. S. L., M. N. P. M., L. M. F., F. R. F. T., D. R. Q. L., and C. H. M. A. performed the histopathological and immunohistochemistry analysis. W. M. S., D. S. C., and M. J. F. performed viral genome sequencing. R. J., V. S. F., L. H. D., A. L. E. A., and E. L. L. A. provided the epidemiological data. D. S. C. and N. R. F. performed the bioinformatics work and assisted with phylogenetic analyses. D. S. C., J. P. C., and N. R. F. performed the statistical analyses. A. R. R. F., J. L. S. G. V., L. T. M. F., L. P. G. C., and F. M. oversaw parts of the laboratory work. J. P. C., O. G. P., L. T. M. F., M. R. T. N., L. P. G. C., and F. M. contributed to the overall design, interpreted results, and commented on article drafts. W. M. S., D. S. C., M. J. F., S. T. S. L., and N. R. F. wrote the article. W. M. S. led the design and execution of the study and oversaw all analyses and interpretation. All authors have seen and approved the final submitted article.

***Acknowledgments.*** The authors thank the staff at the Central Public Health Laboratory of Ceará state, Brazil, for their support and assistance with this investigation. We thank Lucy Matkin for logistic support to the CADDE project and proofreading of the text.

***Disclaimer.*** The funding sources had no involvement in the study.

***Financial support.*** This work was supported by the São Paulo Research Foundation, Brazil (grant numbers 2017/13981-0, 2018/09383-3, 2018/14389-0, 2018/00837-1, and 2019/24251–9); the Brazilian National Council for Scientific and Technological Development (grant number 302584/2015-3, 408338/2018-0); the Clarendon Scholarship; Department of Zoology, University of Oxford; Fundação Cearense de Apoio ao Desenvolvimento Científico e Tecnológico (FUNCAP 01/2017-SUS/PPSUS-CE FUNCAPSESA-Decit/SCTIE); Wellcome Trust and Royal Society Sir Henry Dale Fellowship (grant number 204311/Z/16/Z); and the Network of Clinical and Applied Research into Chikungunya through funds from the Department of Science and Technology, Brazilian Ministry of Health.

***Potential conflicts of interest.*** The authors: No reported conflicts of interest. All authors have submitted the ICMJE Form for Disclosure of Potential Conflicts of Interest.
