# Supplementary material for: Fatal Outcome of Chikungunya Virus Infection in Brazil
Source: Clin Infect Dis. 2020 Aug 7;73(7):e2436–43. doi: 10.1093/cid/ciaa1038 (PMC8492446; doi:10.1093/cid/ciaa1038)
Supplement: ciaa1038_suppl_Supplementary-Appendix [file ciaa1038_suppl_supplementary-appendix.docx]

**Fatal outcome of chikungunya virus infection in Brazil**

Shirlene Telmos Silva de Lima ^1,2,a^, William Marciel de Souza ^3,a,^#, John Washington Cavalcante ^1,a^, Darlan da Silva Candido ^4,a^, Marcilio Jorge Fumagalli ^3,a^, Jean-Paul Carrera ^4,5^, Leda Maria Simões Mello ^2^, Fernanda Montenegro de Carvalho Araújo ^2,6^, Izabel Letícia Cavalcante Ramalho ^2^, Francisca Kalline de Almeida Barreto ^1^, Deborah Nunes de Melo Braga ^7^, Adriana Rocha Simião ^1^, Mayara Jane Miranda da Silva ^8^, Rhaquel de Morais Alves Barbosa Oliveira ^1^, Clayton Pereira Silva Lima ^8^, Camila de Sousa Lins ^6^, Rafael Ribeiro Barata ^8^, Marcelo Nunes Pereira Melo ^6^, Michel Platini Caldas de Souza ^8^, Luciano Monteiro Franco ^6^, Fábio Rocha Fernandes Távora ^6^, Daniele Rocha Queiroz Lemos ^6^, Carlos Henrique Morais de Alencar ^1^, Ronaldo de Jesus ^9^, Vagner de Souza Fonseca ^9,10^, Leonardo Hermes Dutra ^10^, André Luiz de Abreu ^10^, Emerson Luiz Lima Araújo ^10^, André Ricardo Ribas Freitas ^11^, João Lídio da Silva Gonçalves Vianez Júnior ^8^, Oliver G. Pybus ^4^, Luiz Tadeu Moraes Figueiredo ^3^, Nuno Rodrigues Faria ^4,12,b^, Márcio Roberto Teixeira Nunes ^8,b^, Luciano Pamplona de Góes Cavalcanti ^1,b^, and Fabio Miyajima ^1,13,b^.

1. Federal University of Ceará, Fortaleza, Brazil.

2. Central Public Health Laboratory of Ceará State, Fortaleza, Brazil.

3. Virology Research Center, University of São Paulo, Ribeirão Preto, Brazil.

4. Department of Zoology, University of Oxford, Oxford, United Kingdom.

5. Department of Research In Virology and Biotechnology, Gorgas Memorial Institute of Health Studies, Panama City, Panama.

6. Faculdade de Medicina do Centro Universitário Christus, Fortaleza, Ceará, Brazil.

7. Death Verification Service Dr. Rocha Furtado, State Health Secretariat of Ceará, Fortaleza, Ceará, Brazil.

8. Evandro Chagas Institute, Ministry of Health, Ananindeua, Brazil

9. Universidade Federal de Minas Gerais, Brazil.

10. Ministry of Health, Brasilia, Brazil.

11. Faculdade de Medicina São Leopoldo Mandic, Campinas, São Paulo, Brazil.

12. Department of Infectious Disease Epidemiology, Imperial College London, London, UK.

13. Oswaldo Cruz Foundation (Fiocruz), Branch Ceará, Eusebio, Brazil.

^a^S.T.S.L., W.M.S., J.W.C., D.S.C, and M.J.F. contributed equally to this work.

^b^N.R.F., M.R.T.N., L.P.G.C. and F.M. contributed equally to this work.

**#** **Corresponding author:** Dr. William Marciel de Souza, Virology Research Center, University of São Paulo, Av. Bandeirantes, 3900, Monte Alegre, 14090-900, Ribeirão Preto, SP, Brazil. E-mail: [wmarciel@usp.br](mailto:wmarciel@usp.br). Phone: +55 16 3315 4508.

# Supplementary Table 1. Demographic and comorbidities of CHIKV fatal cases (n=68)

| Characteristics | | N (%) |
| --- | --- | --- |
| **Sex** | |  |
|  | Female | 38 (44.1) |
|  | Male | 30 (55.9) |
|  |  |  |
| **Age*** |  | 47.7 ± 24.5 |
|  |  |  |
| **Stage of disease at death (n=68)** | |  |
|  | Acute (0-20 days) | 54 (79.4) |
|  | Sub-acute (>20 days)  Without information | 11 (16.2)  3 (4.4) |
|  |  |  |
|  |  |  |
| **Hypertension (n=41)** | |  |
|  | Yes | 19 (46.3) |
|  | No | 22 (53.7) |
|  |  |  |
| **Heart disease (n=39)** | |  |
|  | Yes | 1 (2.6) |
|  | No | 32 (97.4) |
|  |  |  |
| **DOPC (n=39)** | |  |
|  | Yes | 2 (5.1) |
|  | No | 37 (94.9) |
|  |  |  |
|  |  |  |
| **Asthma** | (n=38) |  |
|  | Yes | 1 (2.6) |
|  | No | 37 (97.4 |
|  |  |  |
| **Kidney failure (n=39)** | |  |
|  | Yes | 1 (2.6) |
|  | No | 38 (97.4) |
|  |  |  |
| **Hypertension and diabetes** | |  |
|  | Yes | 10 (31.25) |
|  | No | 22 (68.8) |
| * Mean ± standard deviation | | |

# Supplementary Table 2. Autopsy findings of 42 chikungunya deaths from Ceará state, Brazil.

| **Brain** | | | |
| --- | --- | --- | --- |
| **Findings** | **Absent** | **Discreet** | **Moderate/ Intense** |
| Congestion | 9.5% (4/42) | 47.6% (20/42) | 42.9% (18/42) |
| Edema | 9.5% (4/42) | 47.6% (20/42) | 42.9% (18/42) |
| Hemorrhage | 78.6% (33/42) | 19.0% (8/42) | 2.4% (1/42) |
| Meningitis | 100% (42/42) | 0% (0/42) | 0% (0/42) |
| Meningoencephalitis | 95.2% (40/42) | 2.4% (1/42) | 2.4% (1/42) |
| Necrosis | 92.8% (39/42) | 2.4% (1/42) | 4.8% (2/42) |
| Thromboembolism | 97.6% (41/42) | 0% (0/42) | 2.4% (1/42) |
| Encephalitis | 78.6% (33/42) | 19.0% (8/42) | 2.4% (1/42) |
| **Lung** | | | |
| **Findings** | **Absent** | **Discreet** | **Moderate/ Intense** |
| Atelectasis | 45.2% (19/42) | 50.0% (21/42) | 4.8% (2/42) |
| Anthracose | 70.7% (29/41) | 29.3% (12/41) | 0% (0/41) |
| Bronchopneumonia | 76.2% (32/42) | 4.8% (2/42) | 19.0% (8/42) |
| Bronchitis | 76.2% (32/42) | 19.0% (8/42) | 4.8% (2/42) |
| Bronchiolitis | 97.6% (41/42) | 2.4% (1/42) | 0% (0/42) |
| Alveolar proteinosis | 9.5% (4/42) | 11.9% (5/42) | 78.6% (33/42) |
| Edema | 7.2% (3/42) | 45.2% (19/42) | 47.6% (20/42) |
| Emphysema | 40.5% (17/42) | 50% (21/42) | 9.5% (4/42) |
| Hemorrhage | 42.9% (18/42) | 42.9% (18/42) | 14.2% (6/42) |
| Pneumonitis | 47.6% (20/42) | 40.5% (17/42) | 11.9% (5/42) |
| Hyaline Membrane | 88.1% (37/42) | 7.1% (3/42) | 4.8% (2/42) |
| Necrosis | 95.1% (39/41) | 4.9% (2/41) | 0% (0/41) |
| Pleuritis | 95.1% (39/41) | 2.4% (1/41) | 2.4% (1/41) |
| Thromboembolism | 100% (42/42) | 0% (0/42) | 0% (0/42) |
| **Heart** | | | |
| **Findings** | **Absent** | **Discreet** | **Moderate/ Intense** |
| Congestion | 7.1% (3/42) | 71.4% (30/42) | 21.4% (9/42) |
| Hemorrhage | 81.0% (34/42) | 19.0% (8/42) | 0% (0/42) |
| Endocarditis | 90.2% (37/42) | 7.3% (3/41) | 2.4% (1/41) |
| Edema | 0% (0/42) | 61.9% (26/42) | 38.1% (16/42) |
| Hypertrophy | 26.2% (11/42) | 52.4% (22/42) | 21.4% (9/42) |
| Necrosis | 90.5% (38/42) | 2.4% (1/42) | 7.1% (3/42) |
| Myocarditis | 63.4% (26/41) | 24.4% (10/41) | 12.2% (5/41) |
| Cardiomegaly | 92.7% (38/41) | 4.9% (2/41) | 2.4% (1/41) |
| **Liver** | | | |
| **Findings** | **Absent** | **Discreet** | **Moderate/ Intense** |
| Colestase | 34.2% (14/41) | 46.3% (19/41) | 19.5% (8/41) |
| Congestion | 9.8% (4/41) | 34.1% (14/41) | 56.1% (23/41) |
| Detrabeculation | 80.5% (33/41) | 12.2% (5/41) | 7.3% (3/41) |
| Edema | 19.5% (8/41) | 46.3% (19/41) | 34.2% (14/41) |
| Steatosis | 41.5% (17/41) | 36.6% (15/41) | 21.9% (9/41) |
| Hemorrhage | 90.2% (37/41) | 9.8% (4/41) | 0% (0/41) |
| Portal infiltrate | 34.2% (14/41) | 46.3% (19/41) | 19.5% (8/41) |
| Necrosis | 78.0% (32/41) | 17.1% (7/41) | 4.9% (2/41) |
| Hepatitis | 41.5% (17/41) | 46.3% (19/41) | 12.2% (5/41) |
| Kupffer cell hypertrophy and hyperplasia | 82.9% (34/41) | 17.1% (7/41) | 0% (0/41) |
| **Spleen** | | | |
| **Findings** | **Absent** | **Discreet** | **Moderate/ Intense** |
| Congestion | 0% (0/37) | 32.4% (12/37) | 67.6% (25/37) |
| Edema | 29.7% (11/37) | 56.8% (21/37) | 13.5% (5/37) |
| Hemorrhage | 43.2% (16/37) | 48.7% (18/37) | 8.1% (3/37) |
| Necrosis | 94.6% (35/37) | 5.4% (2/37) | 0% (0/37) |
| Normal white flesh | 100% (37/37) | 0% (0/37) | 0% (0/37) |
| White pulp hypoplasia | 16.2% (6/37) | 51.4% (19/37) | 32.4% (12/37) |
| White pulp hyperplasia | 94.6% (35/37) | 5.4% (2/37) | 0% (0/37) |
| **Kidney** | | | |
| **Findings** | **Absent** | **Discreet** | **Moderate/ Intense** |
| Congestion | 0% (0/42) | 23.8% (10/42) | 76.2% (32/42) |
| Edema | 7.1% (3/42) | 42.9% (18/42) | 50% (21/42) |
| Hemorrhage | 88.1% (37/42) | 11.9% (5/42) | 0% (0/42) |
| Necrosis | 54.8% (23/42) | 23.8% (10/42) | 21.4% (9/42) |
| Hydropic change | 31% (13/42) | 57.1% (24/42) | 11.9% (5/42) |
| Inflammatory infiltrate | 42.9% (18/42) | 38.1% (16/42) | 19.0% (8/42) |
| Glomerular sclerosis | 59.5% (25/42) | 26.2% (11/42) | 14.3% (6/42) |

#
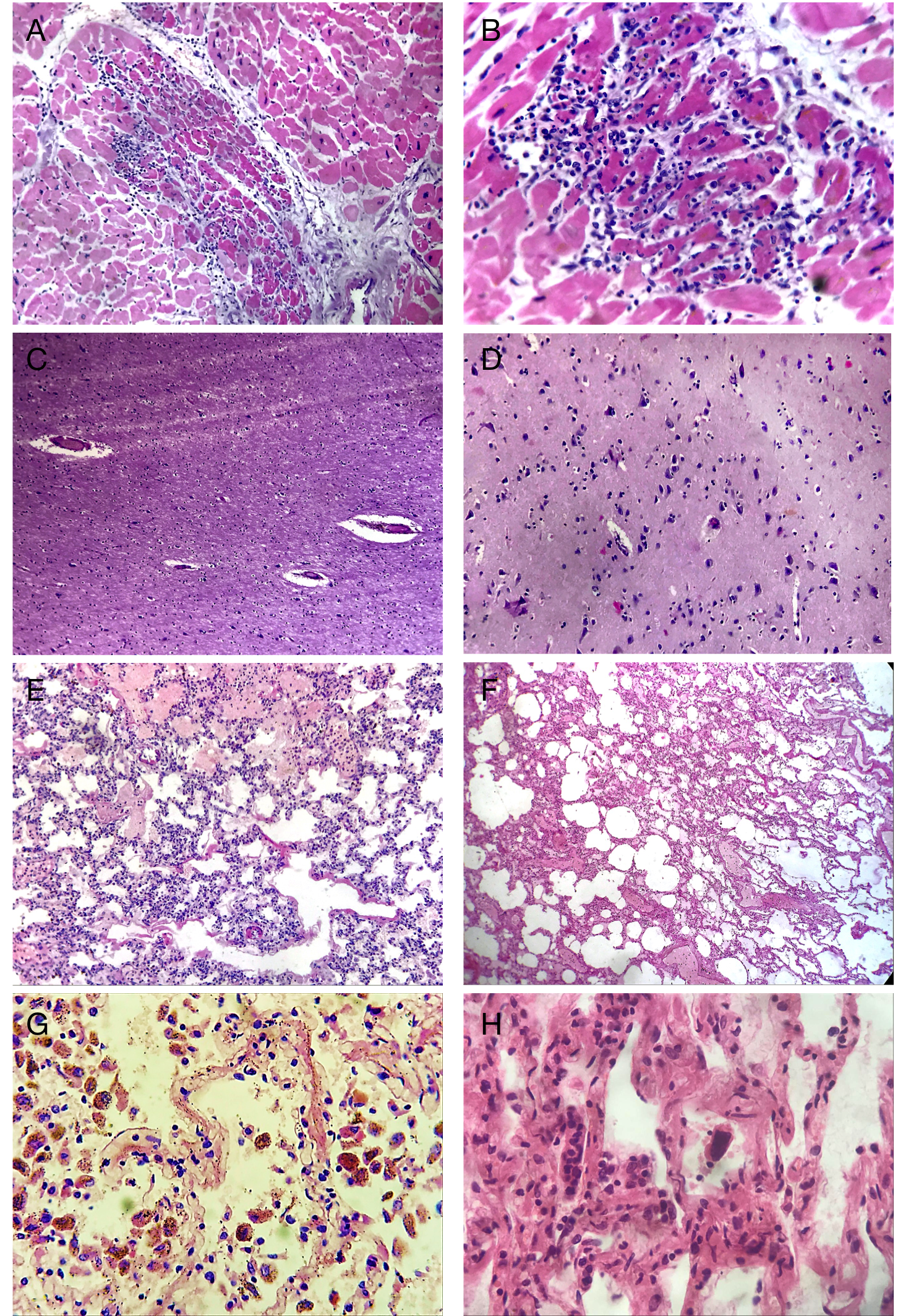
Figure Supplementary 1. Histopathological of chikungunya-deaths in Ceará State. Myocarditis. Mature lymphoplasmacytic infiltrate in cardiac tissue, associated with myocytic necrosis, edema, and neovasal formation. Note myocytes with hypereosinophilia characterizing cellular necrosis (A and B). Encephalitis - Lymphocytic infiltrate in cerebral cortical tissue, associated with reactional gliosis and edema (C and D). Acute bronchopneumonia. Diffuse interstitial infiltrate expanding alveolar septa, with areas of peribronchiolar and perivascular contentment. In some areas, acute interstitial pneumonia pattern and in organization with hyaline membranes (E and F) is observed. (G) Presence of hemosiderophages in lungs of a CHIKV-death. (H) Presence of megakaryocyte in lungs of a CHIKV-death.
